# Negative ion formation and fragmentation upon dissociative electron attachment to the nicotinamide molecule†

**DOI:** 10.1039/d1ra06083j

**Published:** 2021-10-01

**Authors:** Patrick Ziegler, Andrzej Pelc, Eugene Arthur-Baidoo, Joao Ameixa, Milan Ončák, Stephan Denifl

**Affiliations:** Institute for Ion Physics and Applied Physics, University of Innsbruck Technikerstrasse 25 6020 Innsbruck Austria Milan.Oncak@uibk.ac.at Stephan.Denifl@uibk.ac.at; Maria Curie-Skłodowska University, Department of Biophysics, Mass Spectrometry Laboratory Pl. M. C.-Skłodowskiej 1 20-031 Lublin Poland Andrzej.Pelc@poczta.umcs.lublin.pl; Centre of Physics and Technological Research, Departamento de Física, Faculdade de Ciências e Tecnologia, Universidade NOVA de Lisboa 2829-516 Caparica Portugal

## Abstract

Nicotinamide (C_6_H_6_N_2_O) is a biologically relevant molecule. This compound has several important roles related to the anabolic and metabolic processes that take place in living organisms. It is also used as a radiosensitizer in tumor therapy. As a result of the interaction of high-energy radiation with matter, low-energy electrons are also released, which can also interact with other molecules, forming several types of ions. In the present investigation, dissociative electron attachment to C_6_H_6_N_2_O has been studied in a crossed electron-molecular beams experiment in the electron energy range of about 0–15 eV. In the experiment, six anionic species were detected: C_6_H_5_N_2_O^−^, C_5_H_4_N^−^, NCO^−^, O^−^/NH_2_^−^, and CN^−^, with NCO^−^ being the most prominent anion. We also provide detailed computational results regarding the energetic thresholds and pathways of the respective dissociative electron attachment (DEA) channels. The experimental results are compared with the theoretical ones and on this basis, the possible DEA reactions for the formation of anions at a given resonance energy were assigned as well as the generation of neutrals fragments such as pyridine and its several derivatives and radicals are predicted. The pyridine ring seems to stay intact during the DEA process.

## Abstract 2

In this study we show that the biologically relevant nicotinamide molecule dissociates into a variety of fragment anions upon electron attachment. The pyridine ring remains intact in all the dissociation reactions observed *via* mass spectrometry.

## Graphical abstract

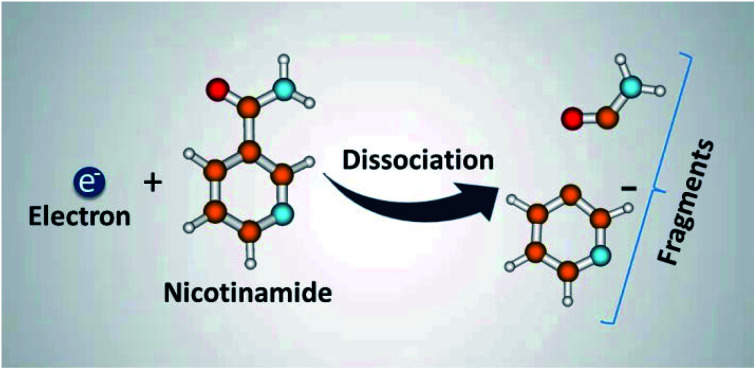

## Introduction

Nicotinamide (NA), also called niacinamide (C_5_H_4_N–CONH_2_, C_6_H_6_N_2_O), is a derivative of the nicotinic acid (called also niacin, C_5_H_4_NCOOH) and is formed by replacing the hydroxyl group in niacin with an amide group. The electrical dipole moment of the NA molecule is 3.315 debye.^1^ It possesses several protonation sites associated with the amide and pyridine (C_5_H_5_N) moieties. Pyridine is also known to be a strong nucleophilic compound, which means that at this site of the NA molecule, there is high electron density. NA may have two possible isomeric structures (*trans*- and *cis*-NA conformers) with changed positions between the oxygen and amide group with respect to the pyridine ring^2^ (in the *cis*-NA isomer, the NH_2_ group is closer to the nitrogen atom in the pyridine ring than the O atom). From the theoretical point of view, the *cis*-NA conformer is more stable with a very small energy difference between both forms of 1 kJ mol^−1^,^3^ which is in the range of the uncertainty of respective calculations.

NA is a compound of great biological relevance. Together with niacin, it is recognized as B3 vitamin or, in other nomenclature, as PP vitamin. This is connected to the fact that it has several functions in living organisms. Most importantly, NA is a part of nicotinamide adenine dinucleotide (NAD^+^ in its oxidized form), which is an essential coenzyme in redox reactions in the energy metabolism.^4^ Moreover, NADH, the reduced version of NAD^+^ formed by the reaction of NAD^+^ with hydride ion, is crucial in several biological processes. Therefore, both NAD^+^ and NADH play serious roles in the metabolic processes and regulate the activity of dehydrogenases, *e.g.*, in glycolysis or fatty acid oxidation. Moreover, NAD^+^ is a substrate in the phosphorylation process that leads to NADP^+^ and NADPH formation. NADP^+^ and NADPH play a role in anabolic processes, *e.g.*, in the biosynthesis of fatty acids and cholesterol.^5^ In the anabolic and metabolic processes, there is electron transfer between molecules involved in the process.^6^

As the NA molecule is an important compound in biological processes, it is also linked to human health. In 1937, Koehn and Elvehjem discovered that NA may be applied in the treatment of pellagra disease (characterized by serious symptoms such as dermatitis, diarrhea, and dementia),^7^ – from which the other name of NA originates (pellagra-preventive (PP) vitamin). This disease is caused by a lack of NA in the diet, which leads to insufficient levels of NAD^+^ and NADP^+^ in the body. Since then, a low level of NA (and the corresponding content of NAD^+^ and NADP^+^) in the body was linked to multiple metabolic and neurodegenerative disease states.^5^ NA also plays a key role in the repair of different types of DNA damages.^5,8,9^ The described functions of NA and derivative compounds also have great influence on the ageing process both in the cell and at the organism level.^10,11^ Besides the use of NA in treating certain health conditions and serving as an agent for delaying aging, NA is also used in the treatment of cancer as a radio- and chemosensitizer.^12–14^ From the point of view of cancer therapy, an effective sensitizer should affect cancer cells much more efficiently than healthy cells. There are two main classes of sensitizers,^15^ namely, (i) hypoxic cell sensitizers and (ii) pyrimidine derivatives, which can be incorporated into the cell DNA due to their nucleoside similarity. The first group interacts with cancer cells as hypoxia is found only in these types of cells. In turn, the activity of the second group is related to the fact that cancer cells undergo rapid and uncontrolled division compared to healthy cells.^15^

It just so happens that NA has properties of both classes of sensitizers; hence, it seems to be a very useful compound in tumor treatment. Studies on the effect of NA on tumor cells showed that NA causes enhanced tumor blood flow, thereby reducing tumor hypoxia and therefore decreasing the presence of radioresistant acutely hypoxic cells.^12^ The effect of oxygen on cancer cells in radiotherapy can be explained as follows: radiation damages DNA by forming free radical sites in DNA. Oxygen is an element with high electronegativity, so it can react with these radicals; hence, the damage of DNA is permanent. In the case of the absence of oxygen, DNA free radicals return to their original form through reactions with H^+^ taken from the non-protein part of the cell. In such a situation, the possibility of destroying a hypoxic cell of the tumor is severely limited.^13^

Another interesting fact regarding NA is that it also has skin photo-protective properties with a very interesting mechanism of action. Here, NA acts as an inhibitor of poly(ADP-ribose) polymerases (PARP); hence, nicotinamide can accelerate DNA repair in cells exposed to UV radiation.^14–17^ Therefore, NA is an ingredient of many lotions that protect the skin against UV radiation, and it is recommended for oral use to increase its activity at the cellular level. The described facts show that the NA functions in the organism are very complex and new research regarding its influence on cells is highly demanded.

High-energy radiation used in cancer therapy may lead to the release of a large number of free low-energy (<100 eV) electrons in cells.^18^ These electrons may then interact with molecules present in the cell as well as with the used drugs (*e.g.*, NA) and induce further damage. Thus, it is very important to know the fragmentation pattern of the NA molecule upon its interaction with an electron and the energetics of such a process. The knowledge of processes occurring during low-energy electron irradiation may then be useful in cancer therapy for the energy and dose of radiation matching.

Because of the abovementioned reasons, it is extremely interesting to study the processes induced by radiation, *e.g.*, the interaction of the NA molecule with low-energy electrons. In the case of positive ion formation from NA, the electron ionization (EI) mass spectrum in the NIST database^19^ shows the formation of several groups of ions in the *m*/*z* (mass to charge ratio) ranges of 16–17, 25–29, 36–45, 61–70, 74–80, 93–95, 103–108, and 122–124. In this spectrum, the main peaks correspond to *m*/*z* of 122 (parent cation), 78 (C_5_NH_4_^+^), 106 (C_6_N_2_H_6_^+^ or C_6_NOH_4_^+^), and 51 (C_4_H_3_^+^). To the best of our knowledge, there is no information or data available regarding the energetics of electron attachment to NA molecules. Such studies are important as it is postulated that the electron attachment process and especially, the associated dissociative electron attachment (DEA), may be the main cause of both actions of some radiation sensitizers^20–22^ and radiation damage in DNA component molecules.^23–25^ DEA studies with other vitamins^26–29^ and coenzyme analogs^26,30^ have been reported previously.

The circumstances described above encouraged us to perform the mass spectrometric investigation of low energy electron interaction with the NA molecule and related theoretical calculations regarding the fragmentation pathways, leading to the generation of the observed anion species.

## Experiment and theory

The electron attachment spectrometer used in the present study comprises a molecular beam source, a high-resolution hemispherical electron monochromator (HEM) and a quadrupole mass filter with a pulse counting system for analyzing and detecting the ionic products. The apparatus has been described previously in detail.^31^ Briefly, the NA sample is in the solid state under room conditions with low vapor pressure of about 0.056 Pa at 298 K, whilst its melting point is 403 K.^32^ For this reason, the NA sample was heated gradually in the resistively heated oven to attain a temperature of 364 K, at which we do not observe thermal decomposition and the ion signal is relatively high. The thermal stability of the sample was checked by the measurement of positive ion mass spectrum at several sample temperatures. The NA vapor was then directly introduced into the interaction chamber of the HEM by a capillary made of copper. The gas flow to the interaction chamber was controlled by the pressure in the main vacuum chamber containing the HEM and the mass spectrometer. In the whole course of the experiment, this pressure was about 4 × 10^−6^ Pa to ensure single-collision conditions. The anions generated by the electron attachment process were extracted by a weak electrostatic field into the quadrupole mass spectrometer, where they were mass-analyzed and detected by a channeltron secondary electron multiplier. After crossing the collision region, the residual electrons were collected by a Faraday plate. The electron current was monitored using a pico-ampere meter.

To determine the energy spread of the HEM and to calibrate the energy scale, the well-known cross section for the formation of Cl^−^/CCl_4_ was used. The formation of Cl^−^/CCl_4_ is characterized by two resonances at 0 eV and about 0.8 eV.^33,34^ The first one was used for the calibration of the electron energy scale and to determine the electron energy spread (the apparent full width at half maximum, FWHM, represents the energy resolution of the electron beam). In the present experiments, the FWHM and the electron current were 100 meV and 19 nA, respectively. The used electron energy resolution represents a reasonable compromise between the product ion intensity and the energy spread to resolve the resonances in the measured ion yields. The HEM was constantly heated to the temperature of 360 K in order to prevent surface charging. The NA sample of 99.8% purity was purchased from Sigma Aldrich, Vienna, Austria.

The nicotinamide molecule and the structures of neutral and anionic fragments were optimized at the B3LYP/aug-cc-pVDZ level and single-point recalculated at the B3LYP/aug-cc-pVTZ level, denoted as B3LYP/aug-cc-pVTZ//B3LYP/aug-cc-pVDZ. For electron affinity calculations, optimization was followed by single-point recalculation at the CCSD(T)/aug-cc-pVTZ level, denoted as CCSD(T)/aug-cc-pVTZ//B3LYP/aug-cc-pVDZ. This approach was chosen based on the benchmarking of small molecules (see Table S1 in the ESI†). In energy difference calculations, zero-point energy correction calculated at the B3LYP/aug-cc-pVDZ level was used. Wavefunction stability was tested for every structure, *i.e.*, it was checked that relaxing various constraints does not lead to lower electronic energies. Vibrational analysis was performed for every stationary point on the potential energy surface to verify its local minimum or first order saddle point character. The electron affinities (EA) of molecules and O atom involved in DEA to NA molecule obtained by the present thermochemical calculation were collected and compared to the experimental values^19,35^ in Table 1. It could be seen that the computed EAs agree well with the experimental ones, with the differences in the range of 0.25 eV and 0.11 eV for B3LYP and CCSD(T) methods, respectively. All calculations were performed in the Gaussian software package.^36^

**Table 1:** Adiabatic electron affinities (EAs, in eV) of the C_6_H_6_N_2_O molecule and relevant neutral fragments as obtained with the B3LYP/aug-cc-pVTZ//B3LYP/aug-cc-pVDZ and CCSD(T)/aug-cc-pVTZ//B3LYP/aug-cc-pVDZ levels and as found in the NIST database^19^ and in ref. 35

Molecule/atom	B3LYP/aug-cc-pVTZ//B3LYP/aug-cc-pVDZ	CCSD(T)/aug-cc-pVTZ//B3LYP/aug-cc-pVDZ	Literature values
C_5_H_4_N–CONH_2_(C_6_H_6_N_2_O)	0.40	0.25	—
C_6_H_5_N_2_O	3.12	3.26	—
C_5_H_4_N	1.40	1.51	1.480 ± 0.006 (ref. 35)
NCO	3.48	3.51	3.6090 ± 0.0050 (ref. 19)
NH_2_	0.76	0.68	0.7710 ± 0.0050 (ref. 19)
CN	4.05	3.87	3.8620 ± 0.0050 (ref. 19)
O	1.68	1.33	1.439157 ± 0.000004 (ref. 19)

## Results and discussion

Negative ions can be formed in several processes, of which the most frequently used in studies of anion generation are (i) direct interaction of a free electron with a molecule,^20,21,24,25,31,34^ (ii) charge transfer between molecules or ions,^37,38^ and (iii) negative thermal ionization.^39,40^ The most valuable data regarding the energetics, energies of electron captured by molecules, and fragmentation routes in the negative ion formation are provided by the mass-spectrometric studies of the direct interactions of an electron with a molecule. In such a process, the electron can be captured by the parent molecule (M), leading to the formation of the so-called temporary negative ion (TNI). Such an ion has an excess of energy (is in excited state), which can lead to electron autodetachment, TNI dissociation (DEA process), or photon emission. The DEA process produces fragment ions (M–N)^−^ and neutral counterparts, marked here generally as N, which can be described by the following equation.

1 M + e^−^ → (M_TNI_)^−^* → (M–N)^−^ + N

More detailed description of the DEA process can be found, for instance, in the work by Feketeová *et al.*^41^ In order to check the anion species formed by electron attachment to NA molecules, multiple mass scans were performed at several incident electron energies in steps of 0.5 eV. For the detected anions, more detailed studies regarding the energetics of the electron capture process, leading to the formation of the measured anions, were performed.

**Fig. 1:**
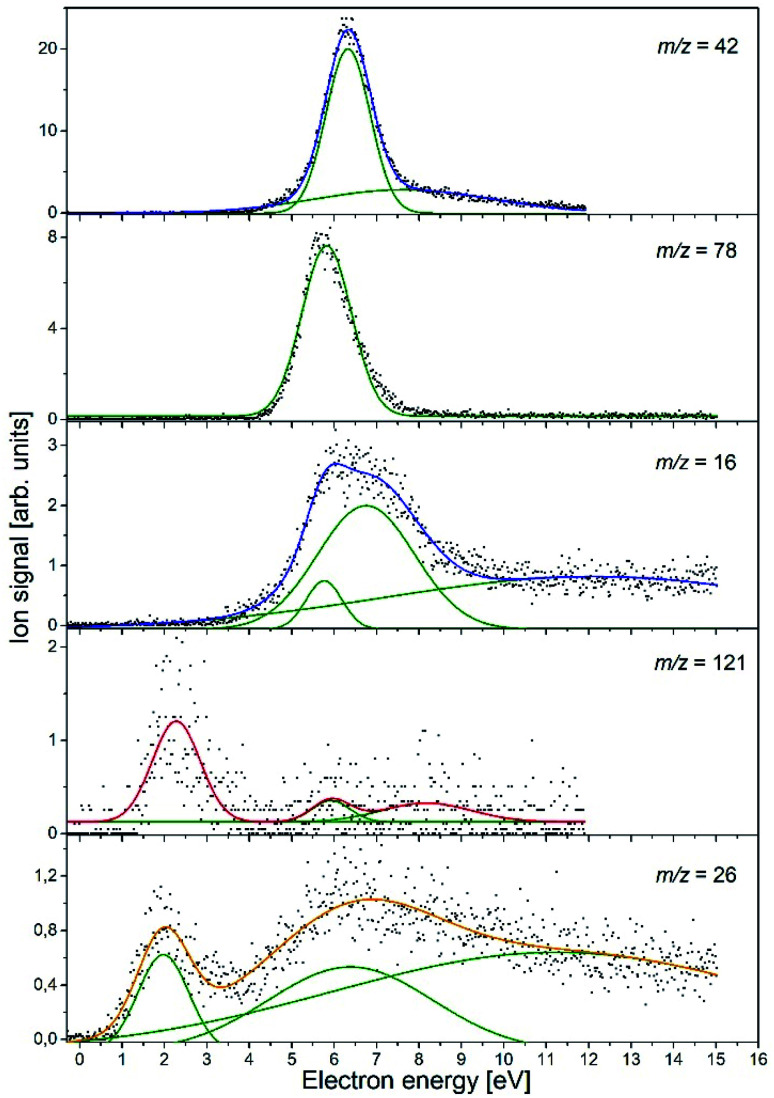
Anion efficiency curves of the fragment anions observed upon DEA to nicotinamide (C_6_H_6_N_2_O). Gaussian peaks fitted to the experimental data, which were then used to estimate the appearance energy and the resonance position, are represented by green lines.

In the course of this electron attachment study with the NA molecule, we observed the generation of the six anionic species with *m*/*z* ratios of 121 (NA–H^−^), 78 (C_5_H_4_N^−^), 42 (NCO^−^), 26 (CN^−^), and 16 (NH_2_^−^ and/or O^−^). In Fig. 1, the anion efficiency curves for all the negatively charged fragments observed for NA in the electron energy range of about 0–15 eV are presented. In Table 2, the peak positions and appearance energies are summarized. The intensities are given in relative units, *i.e.*, the ion signals for all the anions are comparable. The anions are formed mainly at two energy ranges of about 0.5–4 eV and 4–12 eV. At low energy, only two anions were observable, (NA–H)^−^ and CN^−^, whereas the higher energy resonances are characteristic of all the detected anions. The observed anion yields are rather small, which is similar to previous electron attachment studies with pyridine derivative compounds.^42^ It is also worth noting here that low-energy electron interaction with NA is a purely dissociative process. Although the molecule is relatively large and has 39 vibrational degrees of freedom into which the excess energy supplied by the extra electron can be divided, no parent ion was observed in the present experiments. This means that the TNI of the parent molecule is not stable and does not survive longer than a few tens of μs, which is the time needed for the TNI to pass from the ionization chamber to the detector. This observation is in line with previous studies of electron capture by the pyridine and benzene derivatives, in which the parent anion was also not measured.^42–45^ Nevertheless, the EA of the benzene as well as pyridine is negative,^46,47^ whereas the EA of the NA molecule is positive, equal to 0.25–0.40 eV (see Table 1). The EA of NA is close to the EA of 0.45 eV for the other recently investigated molecule, (CH_3_S)_2_CS, for which the parent anion was observed.^48^ Hence, it may be proposed that for NA, the formation of the stable parent anion is possible but due to the low efficiency of this process, the anion yield is below the detection limit of the apparatus. The electron attachment resonance positions were determined by fitting Gaussian peaks to the experimental data, whereas the anion appearance energy (AE) was estimated using the procedure described by Meißner *et al.* in ref. 49, employing the equation AE = *E*_max_ − 2*σ* (where *E*_max_ is the maximum of the Gaussian peak and *σ* is the standard deviation).

**Table 2:** Peak positions of resonances (in eV) with the corresponding appearance energy (AE) mentioned in brackets observed in the ion yield of the fragment anions formed upon dissociative electron attachment to nicotinamide

Ion	Resonance maxima and AE (in parentheses) [eV] (uncertainty ± 0.1 eV)
C_6_H_5_N_2_O^−^	2.3 (1.2), ∼5.9 (∼5.0), ∼8.2 (∼6.0)
C_5_H_4_N^−^	5.8 (4.7)
NCO^−^	6.3 (5.3), 7.6 (3.5)
NH_2_^−^ or O^−^	5.8 (4.9), 6.8(4.5), ∼12.1
CN^−^	2.0 (0.8), 6.4 (2.6), ∼11.2

**Fig. 2:**
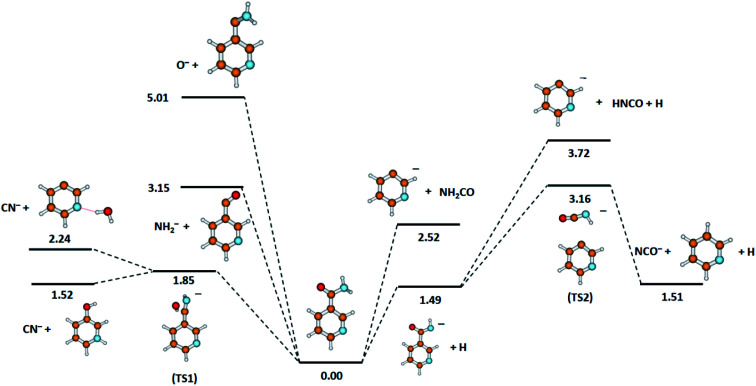
Calculated energies (in eV) for the possible dissociation pathways induced by electron attachment to nicotinamide. Calculated at the B3LYP/aug-cc-pVTZ//B3LYP/aug-cc-pVDZ level of theory.

The calculated DEA pathways for the possible anions detected experimentally, together with the derived thermochemical thresholds for the respective fragmentation routes, are shown in Fig. 2.

### *m*/*z* = 42

The anion with *m*/*z* = 42 is the most abundant anion formed in DEA to the NA molecule. This type of anion may have two structures, namely, NCO^−^ or C_2_H_4_N^−^, which may be formed *via* the following reactions.

2a C_6_H_6_N_2_O + e^−^ → NCO^−^ + C_5_H_6_N

2b C_6_H_6_N_2_O + e^−^ → C_2_H_4_N^−^ + C_4_H_2_NO

where the neutral counterparts occurring in the above DEA channels may also be in different fragmented forms. Both anion species mentioned in reactions (2a) and (2b) were already observed in the previous studies of DEA to different types of molecules.^42,50–52^ Previously, Spisz *et al.* observed the NCO^−^ anion with *m*/*z* = 42 (as well as NSO^−^) in the studies of electron attachment to another radiosensitizer compound, uracyl-5-yl-*O*-sulfamate. This anion was detected at low resonance energy, with experimental thresholds of ∼0 eV.^50^ The formation of such a kind of anion from the NA molecule requires strong molecule fragmentation, breaking the bond between the pyrimidine ring and the CONH_2_ moiety and the simultaneous removal of two hydrogen atoms from the amide group. The generation of the NCO^−^ anion was also mentioned as the only ionic product by Wijeratne and Wenthold^53^ in the collision induced dissociation (CID) studies of the benzoylnitrene radical anion, which is a related compound (C_6_H_5_CON^−^) to the NA molecule. Similarly, NCO^−^ formation was also confirmed in the studies of DEA to acetamide and its derivatives.^54^ The anion with *m*/*z* = 42 was also observed in the studies of the DEA process to the derivatives of pyridine by Ryszka *et al.*^42^ In this investigation on DEA to nicotine and *N*-methyl-pyrrolidine, a structure formula of C_2_H_4_N^−^ was assigned to this anion, which may correspond to three different stoichiometric structures, namely, CHNCH_3_, CHNHCH_2_, and CH_2_CHNH, whereas C_3_H_6_ was excluded from the consideration due to the negative EA. The C_2_H_4_N^−^ anion was measured at high energy (>7 eV) resonances. The generation of anions with such a structure from the NA molecule would require breaking even more bonds in the NA molecule than in the case of NCO^−^ formation, for example, breaking the pyridine ring and removal of the CONH_2_ part from the NA molecule. Moreover, the substantial structure changes related to the reorganization of the bonds of hydrogen atoms would be necessary. In addition, the authors did not observe an anion with the *m*/*z* ratio of 42 in the direct electron attachment to pyridine.^42^ Taking into account the positive ion mass spectra of NA and pyridine,^19^ it can be recognized that in the case of pyridine, the ion with *m*/*z* = 42 is not formed, whereas in the case of the NA molecule, such an ion was detected. Hence, in the case of positive ion formation, the existence of the NH_2_CO moiety in the NA molecule leads to the generation of the ion with *m*/*z* = 42. The abovementioned facts suggest that the NH_2_CO part of the NA molecule is most probably responsible for the formation of the anion with *m*/*z* = 42 in the DEA to NA. Such a statement is additionally supported by the EA values of the considered molecules NCO, CHNCH_3_, CHNHCH_2_, and CH_2_CHNH, which are 3.6 eV (Table 1), 0.55 eV, 0.67 eV, and 1.17 eV,^42^ respectively. Therefore, we may propose that the anion with the structural formula of NCO^−^ corresponds to the measured anion with *m*/*z* = 42.

The experimental data obtained for this anion species (see Fig. 1) show a pronounced resonance peak at higher electron energy regime. The energy of this resonance maximum has a value of 6.3 eV. The peak also has a long tail at the high-energy side, which suggests the existence of another less efficient resonance, leading to the formation of the NCO^−^ ion at a higher energy range. More accurate analysis of the NCO^−^ yield indicates a second resonance centered at 7.6 eV. The appearance energies for both resonances derived in our studies are 5.3 eV and 3.5 eV, respectively.

Considering the results of the thermochemical calculations, it seems that the formation of the NCO^−^ anion involves a few NA rearrangements (see Fig. 2). The formation of NCO^−^ may be described by the following subsequent reactions.

2c C_6_H_6_N_2_O + e^−^ → C_6_H_5_N_2_O^−^ + H (Δ*E* = 1.49 eV)

2d C_6_H_5_N_2_O^−^ → [C_5_H_4_N⋯NHCO]* (transition state TS2, Δ*E* = 1.67 eV)

2e [C_5_H_4_N⋯NHCO]* → NCO^−^ + C_5_H_5_N (Δ*E* = −1.65 eV)

where the respective reaction thermochemical energy threshold (Δ*E*) is provided in parenthesis for every step. In the first step, the cleavage of the hydrogen bond in the amine group proceeds (2c) at the electron energy threshold of 1.49 eV. This anion (NA–H)^−^ then enters the TS2 transition state (2d) with a fairly large energy barrier of 1.67 eV (3.16 eV with respect to NA). In the TS2 state, the bond between the NHCO^−^ moiety and the pyridine ring is broken. In the final step, the NHCO^−^ fragment loses the hydrogen atom, forming the NCO^−^ anion. The released H atom creates a new bond with the ring, with the result that a neutral pyridine molecule is formed (2e). The highest energy barrier to overcome for NCO^−^ formation (corresponding to the formation of TS2) is lower than both the AEs of the resonances observed in our experimental studies. Therefore, we propose that the resonance with AE = 3.5 eV may correspond to the two-stage dissociation process described by the reactions (2c) and (2e). The second resonance may be then assigned to the DEA *via* the excited state of the molecule or to the DEA involving additional fragmentation of the neutral counterpart (C_5_H_5_N) formed in the reaction (2e). It should also be mentioned that in the case of benzamide, another three-stage process leading to NCO^−^ generation was predicted.^53^ There, in the first two stages, both hydrogen bonds in the amide group are disrupted and then the bond between the pyridine ring and the remaining NCO^−^ moiety is cleaved.

### *m*/*z* = 78

The anion with *m*/*z* = 78 is the second most abundant negatively charged species in the case of DEA to the NA molecule. The formation of this type of anion from the NA molecule may be directly related to the generation of the pyridine ring anion in agreement with the following reaction.

3a C_6_H_6_N_2_O + e^−^ → C_5_H_4_N^−^ + NH_2_CO (Δ*E* = 2.52 eV)

Reaction (3a) involves single bond cleavage between the pyridine ring and the NH_2_CO group. It is also worthy to note that the C_5_H_4_N fragment possesses relatively low value of EA of 1.48 eV (see Table 1), which may explain its low efficiency of formation in the process of DEA to the NA molecule.

Quantum chemical calculations indicate that another pathway of C_5_H_4_N^−^ anion generation is also possible (see Fig. 2). This second route of C_5_H_4_N^−^ formation would be a two-stage process. Firstly, the TNI of the NA molecule dissociates to the (NA–H)^−^ anion according to reaction (2c) and then fragmentation proceeds, as described by the following reaction.

3b C_6_H_5_N_2_O^−^ → C_5_H_4_N^−^ + NHCO (Δ*E* = 2.23 eV)

Such ring anion formation is generally characteristic of electron attachment to aromatic compounds, for *e.g.*, benzene and pyridine as well as their derivatives.^42,43,45,47,55–57^ For instance, the generation of the C_5_H_4_N^−^ anion (*m*/*z* = 78) was also confirmed in the previous study of DEA to pyridine,^42^ indicating three pronounced resonances at 2.5 eV, 5.3 eV, and 9.0 eV. Similarly, in close connection to the radiosensitizing properties of NA, such a formation of anionic species was also predicted in the studies of γ radiolysis by Grimison *et al.*^55^

The C_5_H_4_N^−^ anion yield shown in Fig. 1 indicates that this anion is formed most likely *via* a core-excited resonance at energies in the range of about 4 to 9 eV. The resonance peak is centered at 5.8 eV and the AE value is estimated to be 4.7 eV. With such AE, both channels (3a) and (3b) are accessible. Considering that the DEA channel (2c) and (3b) requires about 1.2 eV more energy than the process described by reaction (3a) and, in addition, that channel (3b) competes with the energetically favorable reactions (2d) and (2e) leading to the formation of NCO^−^, the formation of C_5_H_4_N^−^ at the resonance peak of 5.8 eV is most likely associated with the DEA described by reaction (3a). In the plot of the C_5_H_4_N^−^ ion signal *versus* the electron energy, a small ion signal might be seen, which is the tail of the main resonance peak for the higher energies. This signal could also come from another resonance, which could relate to this ion formation through the channel (3b). However, due to the very low signal, this assumption can only be treated as a hypothesis.

### *m*/*z* = 16

The formation of the anion with *m*/*z* = 16 from the NA molecule may correspond to the generation of three isobaric anionic species, namely, NH_2_^−^, O^−^ and/or CH_4_^−^. The latter may be excluded from further consideration as its formation would require the complex reorganization of the NA molecule. In turn, due to anti-cancer therapy application, it would be most beneficial to form the O^−^ anion by DEA to NA, which could have some influence on the hypoxic cells of the tumor. The EAs of the oxygen atom and the amide group are 1.44 eV and 0.77 eV, respectively, indicating that from the EA point of view, the generation of O^−^ ion is more favorable.

Computational results (Fig. 2) show that both types of anions can be formed by simple bond cleavage in the H_2_NCO moiety. DEA channels leading to the formation of NH_2_^−^ and O^−^ anions in the case of NA may be expressed by the following reaction pathways.

4a C_6_H_6_N_2_O + e^−^ → NH_2_^−^ + C_5_H_4_NCO (Δ*E* = 3.15 eV)

4b C_6_H_6_N_2_O + e^−^ → O^−^ + C_5_H_4_NCNH_2_ (Δ*E* = 5.01 eV)

Both reactions are endothermic and due to the higher dissociation energy of the C=O bond (*ca.* 5.5 eV) with respect to C–NH_2_ (about 3.7 eV),^58^ reaction (4b) leading to the formation of O^−^ requires almost 1.9 eV more energy than the formation of the NH_2_^−^ anion by reaction (4a).

The anion efficiency curve for the anion with *m*/*z* = 16 shows a broad feature in the energy range of about 4 to 9 eV with a distinct tail extending to the end of the measured electron energy range. A thorough analysis of the ion signal indicates the existence of three resonances, leading to the generation of the anion. These recognized resonances are centered at 5.8 eV, 6.8 eV, and ∼12.1 eV. The experimental AE of the respective resonances are 4.9 eV and 4.5 eV for the lower energy resonances, while the experimental formation threshold of the anion with *m*/*z* = 16 in the case of resonance at 12.1 eV is burdened with high inaccuracy due to a very low anion signal. Comparing the experimental (for *m*/*z* = 16) and theoretical values of the thresholds for the generation of NH_2_^−^ and O^−^, it can be proposed that both anion species may be formed in accordance to the DEA channel (4a) and (4b) depending on the amount of additional energy (kinetic and excitation) accumulated in the resulting fragments. It is also worthy to note that in the previous studies of DEA to benzaldehyde, with the C=O group similar to that in NA, O^−^ anion generation was also observed at three higher energy (7.8, 8.9, and 10.5 eV) resonances.^59^ The *m*/*z* = 16 anion was also measured in the investigations of electron capture by acetamide (CH_3_CONH_2_).^60^ Abdoul-Carime *et al.* assigned the ion with *m*/*z* = 16 to O^−^ based on earlier observations, which indicated the lack of formation of the NH_2_^−^ ion from the molecules containing the amide group. However, as recent studies show, the formation of NH_2_^−^ from molecules having the NH_2_ moiety is quite an efficient process.^61,62^ Definitely more insight into the formation of the structure of the anion with *m*/*z* = 16 was brought by studies of electron capture by the glycine molecules using high-resolution mass spectrometry.^58^ These investigations showed that both types of ions, *i.e.*, O^−^ and NH_2_^−^, are formed in the interaction of low-energy electrons with glycine molecules. It was also observed that at lower electron energies (∼6.5 eV), NH_2_^−^ ions are generated, while the DEA channel leading to the formation of O^−^ requires higher electron energies (∼11.5 eV). The identical conclusions regarding the formation of O^−^ and NH_2_^−^ anions and the respective energy requirements were drawn from the DEA studies in the case of formamide.^63^ Based on the previous results for the similar structured molecules, we propose that most likely, the low energy resonance (5.8 eV) corresponds to NH_2_^−^ anion generation, whilst the formation of O^−^ proceeds at higher energies. This energetic order of the generation of O^−^ and NH_2_^−^ anions also agrees with the energetic thermochemical thresholds for the DEA channels involved (4a) and (4b). Nevertheless, the exact determination of the anion formed at a given resonance energy requires additional research using isotope substitution in the molecule.

### *m*/*z* = 121

The generation of the anion with *m*/*z* = 121 directly corresponds to the single hydrogen bond cleavage in the NA molecule. The loss of one hydrogen atom is a common DEA channel leading to the formation of the (M–H)^−^ anion, which was observed for several organic molecules.^54,60–62,64^ The formation of the dehydrogenated parent anion from the NA molecule is an endothermic process described by the following DEA reaction.

5 C_6_H_6_N_2_O + e^−^ → C_6_H_5_N_2_O^−^ + H (Δ*E* = 1.49 eV)

**Fig. 3:**
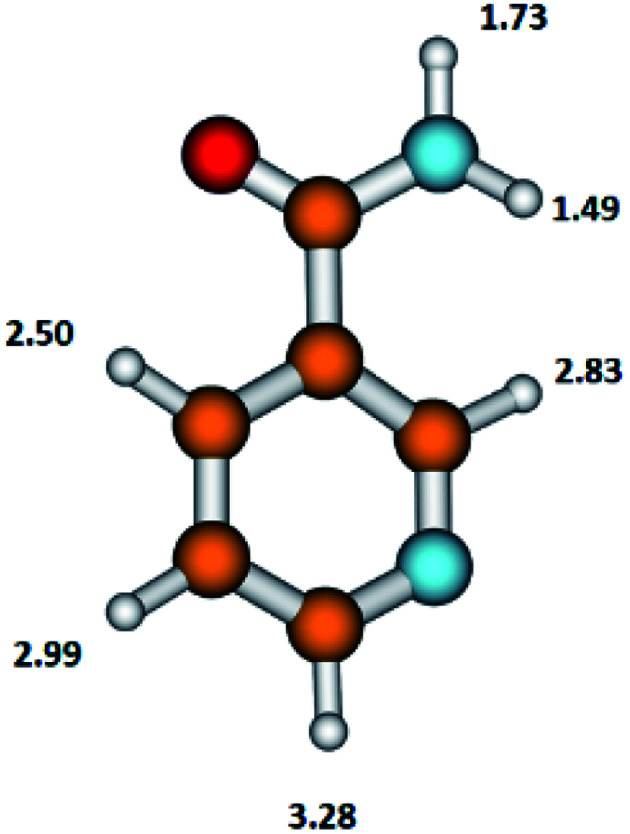
The structure of the nicotinamide molecule with marked thermochemical thresholds (in eV) on the formation of the (M–H)^−^ anion by respective H atom abstraction according to reaction (5). Calculated at the B3LYP/aug-cc-pVTZ//B3LYP/aug-cc-pVDZ level of theory.

The NA molecule has six different sites for H abstraction (in the pyridine ring and in the NH_2_CO moiety); therefore, the detachment of the H atom from the parent molecule leading to the formation of the (NA–H)^−^ anion may occur at different energies. The presently calculated energy thresholds for the formation of the (M–H)^−^ anion associated with the breaking of the bond with the H atom in individual positions in the NA molecule are shown in Fig. 3.

These results show that in the formation of the C_6_H_5_N_2_O^−^ anion, the disruption of the N–H bond in the NH_2_CO moiety of the NA molecule with the thresholds of 1.49 or 1.73 eV is energetically favorable. The DEA threshold for C–H bond disruption in the pyridine ring requires almost double the amount of energy (in the range from 2.50 to 3.28 eV) compared to the N–H bond cleavage. The energetics of this process shows that the pyridine ring moiety is more stable upon the DEA process compared to the NH_2_CO moiety.

The C_6_H_5_N_2_O^−^ yield shown in Fig. 1 indicates that this anion is formed with surprisingly low intensity but in a large range of energy of about 1 to 12 eV. In this ion yield curve, one can distinguish a distinct resonance with the maximum at 2.3 eV. Two other poorly resolved resonances with centers at ∼5.9 and ∼8.2 eV can be fitted to the signal at higher energies. The respective AEs of all the mentioned resonances are 1.2, ∼5, and ∼6 eV, respectively. The experimental AE of C_6_H_5_N_2_O^−^ is therefore lower by 0.3 eV than its calculated thermochemical threshold. This might be rationalized by the thermal excitation of the parent anion, which could shift the energy needed for dissociating the hydrogen atom to lower values. It can be thus concluded that anion formation at the resonance peak of 2.3 eV is related to the breaking of the one N–H bond in the amide group. The observed low intensity signal at higher energies would then correspond to another bond cleavage in the NH_2_ moiety (with a threshold of 1.73 eV) or to the disruption of the C–H bond in the pyridine ring's part of the NA molecule.

The previous results of the DEA studies of pyridine are also not very helpful in supporting the above assignments.^42^ In that case, the formation of the (M–H)^−^ ion was observed at low energy (resonance at 2.5 eV) as well as at higher energies (resonances at 5.3 and 9.0 eV). A similar situation repeats itself in the case of studies of the (M–H)^−^ anion from benzamide, where only N–H bond disruption was considered to be involved in the formation of this anion.^53^

### *m*/*z* = 26

The anion at *m*/*z* = 26 may be related to the formation of two isobaric species of C_2_H_2_^−^ and CN^−^. The generation of C_2_H_2_^−^ with the vinylidene structure of C=CH_2_^−^ was observed previously in the studies of DEA to glycine.^58^ C=CH_2_^−^ was formed in a high-energy resonance of about 11 eV with an energetic threshold of 7.6 eV. It is also worth adding here that the C=CH_2_^−^ anion resulting from glycine (H_2_NCH_2_COOH) may be formed by direct bond cleavage, whereas in the case of the NA molecule, the formation of the C=CH_2_ radical involves substantial molecular fragmentation and rearrangement. This fact together with the low EA of C=CH_2_ (0.48 eV (ref. 19)) would lead to the generation of the C=CH_2_^−^ anion at probably quite high electron energies, exceeding those observed for glycine in ref. 58. In contrast, our experimental data indicate that an anion with *m*/*z* = 26 is formed with two distinct resonances centered at 2.0 and 6.4 eV. The second resonance has a long tail; therefore, we carried out a more detailed analysis of this ionic signal, which confirmed the existence of an additional resonance peak at ∼11.2 eV. The derived AEs for the apparent (lower-energy) resonances are 0.8 and 2.6 eV. The high-energy resonance has the AE below 4 eV (we will not provide here an exact value due to the high inaccuracy of the peak position estimation related to the very low signal). These facts rather exclude the possibility of formation of the anion with *m*/*z* = 26 in the C=CH_2_^−^ structure. Instead, the anion at *m*/*z* = 26, formed in DEA to NA molecule, has most likely the structure of CN^−^. The CN species is often called a pseudohalogen molecule as it possesses a huge value of EA of about 3.86 eV (Table 1), which is even higher than the EA of the halogen atoms. The CN^−^ anion was detected in several studies of electron attachment to the nitrile,^62,65^ amino,^58,61^ as well as nitro^66,67^ compounds. Despite the huge EA of the CN molecule, the corresponding anion signals observed in previous studies was relatively low. Also, in the present study, CN^−^ anion formation has a very low efficiency. The CN^−^ signal has the lowest intensity among all the detected anionic species. This aspect of CN^−^ formation has been explained previously based on the study of electron attachment to various nitrile molecules^68^ and corresponds to the changes in the molecule structure and its symmetry.

Considering our quantum chemical calculations (see Fig. 2), two DEA channels leading to CN^−^ formation from the NA molecule may be derived, in accordance with the following reactions.

6a C_6_H_6_N_2_O + e^−^ → CN^−^ + C_5_H_6_NO (Δ*E* = 1.52 eV)

6b C_6_H_6_N_2_O + e^−^ → CN^−^ + C_5_H_4_N⋯H_2_O (Δ*E* = 2.24 eV)

**Fig. 4:**
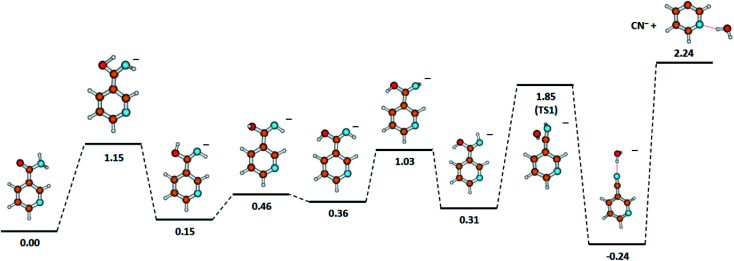
The proposed pathway of CN^−^ formation with marked energetic barriers (in eV). Calculated at the B3LYP/aug-cc-pVTZ//B3LYP/aug-cc-pVDZ level of theory.

One detailed pathway leading to CN^−^ generation is presented in Fig. 4. CN^−^ formation is related to several changes in the configuration of the NA molecule; hence, several potential barriers must also be overcome. Both DEA channels (6a) and (6b) process through a transition state (TS1) with an energy barrier of 1.85 eV, which is higher than the thermochemical threshold for the reaction (6a); however, further barriers might be expected for completing pathway (6a). The barrier of 1.85 eV is significantly higher (*ca.* 1 eV) than the experimentally obtained AE for the first resonance peak at 2.0 eV but below the peak maximum. To explain the formation of the CN^−^ anion at low energies, the following possibilities may be considered: (i) different dissociation pattern of CN^−^ formation, (ii) DEA reactions to vibrationally excited states of the molecule, and (iii) electron attachment to an impurity of the sample. Regarding the first option, the other possible DEA channel of CN^−^ formation would involve the fragmentation of the pyridine ring. This hypothesis may be rejected as the *m*/*z* = 26 anion was not observed in the earlier study of electron capture by pyridine molecules.^42^ The second possibility, similarly, seems to be very unlikely due to the relatively low temperature of the NA sample during the measurements, which excludes the possibility of delivering about 1 eV to the molecule. The last route of the formation of CN^−^ seems to be the most probable. Firstly, the peak at about 2 eV is observed at almost all the CN^−^ signals measured for several compounds.^58,61,62,65^ Also, the signal intensity is very low (about 1 cps), while for the other molecules studied, this anion is always one of the most effectively generated anionic species. Taking this assumption under consideration, only the resonances observed at higher energies may therefore be assigned to the reaction channels described by reactions (6a) and (6b).

## Conclusion

Nicotinamide is a molecule of special importance in several processes occurring in the living organisms and is also considered as a sensitizing compound in cancer radiotherapy. The resistance of a nicotinamide molecule to ionizing radiation is hence an especially important property with respect to cancer treatment. Herein, we studied dissociative electron attachment to nicotinamide in the gas phase using electron attachment spectroscopy. Our study has shown that electron capture by the NA molecule is not an efficient process. We detected six anionic species, the ones with the highest yields were NCO^−^ and C_5_H_4_N^−^ (pyridine ring from NA molecule). The anions are formed in two energy ranges of about 0.5–4 eV and 4–12 eV. One of the major observations in the present DEA studies to NA is that all the anions formed result directly from the bond disruption in the H_2_NCO moiety, and no bond cleavage in the pyridine ring was detected in the studied electron energy range. Moreover, in the DEA channels, pyridine-related radicals (*e.g.*, C_5_H_6_NO˙ and C_5_H_4_N˙) were generated. This is of special importance as the radiation can cause DNA damage and radicals, for *e.g.*, resulting from the electron–molecule interaction, can be then incorporated into the damaged DNA structures. This process is effective for species such as pyridine that has a similar structure to the nucleobases, especially if we consider its nucleophilic properties. In this way, the change in the genetic code of the tumor cell proceeds, hindering its transcription, and consequently leading to cell death, hence also enhancing the therapeutic effect of other drugs used in the medical treatment. We also observed the O^−^ radical anion, which may have some influence on the treated cancer hypoxic cells and may also be incorporated into the damaged DNA structure.

## Conflicts of interest

There are no conflicts to declare.

## Supplementary Material

RA-011-D1RA06083J-s001
