# Supplementary material for: Negative ion formation and fragmentation upon dissociative electron attachment to the nicotinamide molecule
Source: RSC Adv. 2021 Oct 1;11(51):32425–34. doi: 10.1039/d1ra06083j (PMC9041917; doi:10.1039/d1ra06083j)
Supplement: RA-011-D1RA06083J-s001 [file RA-011-D1RA06083J-s001.pdf]

Electronic Supplementary Material (ESI) for RSC Advances.
This journal is © The Royal Society of Chemistry 2021

Supporting Information

for

Negative ion formation and fragmentation upon dissociative electron attachment to the nicotinamide molecule

Patrick Ch. Ziegler[1], Andrzej Pelc[*2], Eugene Arthur-Baidoo[1], Joao Ameixa[1,3], Milan Ončák[1], Stephan Denifl[*1]

[1]Institute for Ion Physics and Applied Physics, University of Innsbruck, Technikerstrasse 25, 6020 Innsbruck, Austria
[2]Maria Curie-Skłodowska University, Department of Biophysics,
Mass Spectrometry Laboratory, Pl. M. C.-Skłodowskiej 1, 20-031 Lublin, Poland
[3]Centre of Physics and Technological Research, Departamento de Física, Faculdade de Ciências e Tecnologia, Universidade NOVA de Lisboa, 2829-516 Caparica, Portugal
**e-mail: Andrzej.Pelc@poczta.umcs.lublin.pl, Stephan.Denifl@uibk.ac.at*

Table S1 – Electron affinities (in eV) for four selected molecules as optimized at the B3LYP/aug-cc-pVDZ level and single-point recalculated using various methods. The zero-point energy correction was included as calculated at the B3LYP/aug-cc-pVDZ level. Mean absolute error with respect to experimental values is also given ("Error").

molecule	B3LYP/aug-cc-pVDZ	B3LYP/aug-cc-pVTZ	CCSD(T)/aug-cc-pVDZ	CCSD(T)/aug-cc-pVTZ
CN	4.04	4.05	3.74	3.87
NCO	3.48	3.48	3.37	3.51
NH2	0.75	0.76	0.55	0.68
O	1.68	1.68	1.19	1.33
Error	0.14	0.14	0.21	0.08

Cartesian coordinates (in Å) and electronic energies (in Hartree, including zero-point correction) of structures optimized at the B3LYP/aug-cc-pVDZ level

```
NAD
E = -416.933556
C 0.207108 0.032520 -0.005485
C -0.493904 -1.177697 -0.120759
N -1.829191 -1.272172 -0.114346
C -2.526925 -0.135228 0.008057
C -1.932058 1.127040 0.113976
C -0.542624 1.211717 0.101817
H 0.036724 -2.124239 -0.249663
H -2.550609 2.020120 0.201507
H -0.020510 2.165098 0.170555
C 1.706895 0.143522 -0.022825
```

O 2.265729 1.215998 -0.225035
N 2.415296 -1.011488 0.177454
H 1.988941 -1.849775 0.539922
H 3.420553 -0.927592 0.230218
H -3.614614 -0.237218 0.017295

NAD-
E = -416.949429
C 0.240094 0.059557 -0.021495
C -0.491030 -1.178968 -0.056820
N -1.809811 -1.298001 -0.031674
C -2.555368 -0.152567 0.023458
C -1.952974 1.126377 0.039528
C -0.576323 1.250792 0.015020
H 0.057275 -2.124625 -0.131365
H -2.586847 2.018029 0.070102
H -0.084675 2.222791 0.024752
C 1.670077 0.153352 -0.018407
O 2.322018 1.232195 -0.042581
N 2.421033 -1.070190 -0.021231
H 2.021400 -1.810977 0.549566
H 3.372058 -0.863471 0.268340
H -3.640775 -0.273221 0.041877

[NAD-O]-
E = -341.613098
C -0.420378 1.307245 -0.034616
C 0.515753 0.264814 -0.171326
C 0.006628 -1.053165 -0.190870
N -1.288415 -1.362350 -0.057647
C -2.150176 -0.353161 0.124171
C -1.764623 0.993415 0.140755
C 1.937673 0.577707 -0.374365
N 2.742487 -0.205492 0.310077
H 0.679449 -1.895637 -0.372549
H -2.512992 1.775459 0.270647
H -0.076533 2.341509 -0.062559
H 2.435296 -0.930572 0.970177
H 3.746026 -0.106479 0.212397
H -3.199012 -0.630525 0.252384

O-
E = -75.138730
O 0.000000 0.000000 0.000000

[NAD-NH2]
E = -360.917050
C 0.372197 1.304918 0.000072
C -0.547217 0.249273 0.000137
C -0.058037 -1.072774 0.000171
N 1.242689 -1.369303 -0.000068
C 2.112390 -0.345416 0.000043
C 1.732387 1.000608 -0.000087
C -2.003531 0.526825 0.000021
O -2.907799 -0.248581 -0.000118
H -0.759850 -1.909098 -0.000240
H 2.488424 1.785225 -0.000343
H 0.015623 2.335192 0.000020
H 3.170235 -0.618149 -0.000161

NH2-
E = -55.899592
N 0.000000 -0.000000 0.144578
H -0.000000 0.804290 -0.506022
H -0.000000 -0.804290 -0.506022

CN-
E = -92.868847
N 0.000000 0.000000 0.546168
C 0.000000 0.000000 -0.637196

NCO-
E = -168.147819
N 0.000000 0.000000 -1.268062
C 0.000000 0.000000 -0.070712
O 0.000000 0.000000 1.162588

C5H4N-
E = -247.603805
C -1.137354 -0.800245 0.000000
C 0.082463 -1.523321 -0.000000
C 1.193855 -0.636942 -0.000000
N 1.200647 0.720660 -0.000000
C -0.000000 1.321825 0.000000
C -1.199299 0.604206 0.000000
H 2.211923 -1.056901 -0.000000
H -2.155802 1.143232 0.000000
H -2.096363 -1.343385 0.000000
H -0.002279 2.419304 0.000000

CONH2
E = -169.236215
C 0.160201 -0.411694 0.000507
O 1.218584 0.153685 -0.000209
N -1.084071 0.094824 -0.000344
H -1.879503 -0.523101 -0.000366
H -1.241881 1.100021 0.001405

CONH
E = -168.689093
C -0.000000 0.050769 0.000000
O -0.571200 1.073550 -0.000000
N 0.447629 -1.085113 0.000000
H 1.436197 -1.297219 0.000000

H
E = -0.501657
H 0.000000 0.000000 0.000000

[NAD-H]-, iso1
E = -416.370413
C 1.138151 -0.546246 0.000000
C -0.000000 0.272188 0.000000
C -1.238466 -0.393118 0.000000
N -1.401001 -1.728399 0.000000
C -0.289352 -2.479717 0.000000
C 0.997357 -1.935981 0.000000
C 0.198127 1.810445 0.000000
N -0.852391 2.615817 0.000000
O 1.409779 2.172125 0.000000
H -2.165728 0.184568 0.000000
H 1.869347 -2.594408 0.000000

H 2.106971 -0.046136 0.000000
H -1.712879 2.066001 0.000000
H -0.437096 -3.564377 0.000000

[NAD-H]-, iso2
E = -416.331076
C -0.550357 1.223574 0.019718
C 0.195834 0.041866 0.004739
C -0.397262 -1.266603 -0.016809
N -1.776957 -1.311850 -0.018297
C -2.489502 -0.173407 -0.005486
C -1.942732 1.115903 0.013535
C 1.696051 0.131684 -0.000044
N 2.283948 -1.094568 0.046250
O 2.338464 1.194209 -0.037301
H -2.590356 1.996210 0.024107
H -0.047437 2.194009 0.035261
H 1.607550 -1.873740 0.005264
H 3.284400 -1.174492 -0.046273
H -3.582998 -0.288835 -0.009541

[NAD-H]-, iso3
E = -416.314376
C -0.614152 1.222513 0.030991
C 0.172869 0.053363 -0.019374
C -0.539775 -1.161501 -0.071286
N -1.870966 -1.283951 -0.049076
C -2.697636 -0.163344 0.032272
C -1.996254 1.093855 0.051007
C 1.647594 0.153848 -0.014449
N 2.364471 -1.037637 0.044389
O 2.268786 1.220570 -0.083785
H 0.009136 -2.111855 -0.154979
H -2.598940 2.009155 0.097642
H -0.112796 2.193620 0.062575
H 1.914241 -1.870145 0.395622
H 3.347663 -0.926622 0.247260

[NAD-H]-, iso4
E = -416.324893
C -0.518798 1.226259 -0.102343
C 0.169450 -0.007474 -0.011278
C -0.611341 -1.172936 0.066862
N -1.948309 -1.149298 0.079127
C -2.537638 0.073227 0.019096
C -1.925061 1.350149 -0.085555
C 1.652189 -0.150439 -0.013436
N 2.379165 1.006822 0.193335
O 2.248856 -1.222882 -0.161724
H -0.121159 -2.147138 0.127147
H 0.079505 2.145805 -0.216201
H 1.925475 1.907194 0.159503
H 3.371771 0.952665 0.020645
H -3.635239 0.009156 0.055389

[NAD-H]-, iso5
E = -416.343116
C 0.395048 -1.297197 -0.014384
C -0.193365 0.001134 0.002348
C 0.563249 1.183897 0.015931
N 1.906149 1.205452 0.014057
C 2.501096 -0.003566 -0.002210
C 1.809760 -1.219350 -0.015126
C -1.694178 0.121231 -0.000826
N -2.307282 -1.094941 0.040057
O -2.323453 1.190643 -0.031926
H 0.058566 2.154387 0.028979
H 2.417207 -2.135591 -0.026983
H -1.660277 -1.891608 0.003551
H -3.309790 -1.153908 -0.038121
H 3.600187 0.011097 -0.005217

[NAD-H]-, iso6
E = -416.379452
C 1.156475 -1.864545 0.000000
C 1.197471 -0.466416 0.000000
C -0.000000 0.256435 -0.000000
C -1.194136 -0.487682 0.000000
N -1.254591 -1.830808 0.000000
C -0.088016 -2.497626 0.000000
C 0.029653 1.785565 -0.000000
O 1.182550 2.320515 -0.000000
N -1.169059 2.347467 -0.000000
H -2.138886 0.056053 -0.000000
H 2.072954 -2.459517 0.000000
H 2.130283 0.098170 -0.000000
H -1.013128 3.360787 -0.000000
H -0.154753 -3.590621 0.000000

[NAD-CNOH]
E = -248.225812
C 1.200027 0.673556 0.000057
C 0.000299 1.387814 0.000016
C -1.199771 0.673792 -0.000125
C -1.144870 -0.723691 0.000175
N -0.000140 -1.421262 -0.000068
C 1.144440 -0.724223 -0.000035
H -2.162889 1.185183 0.000077
H -2.066324 -1.311113 -0.000124
H 2.163226 1.184320 -0.000033
H 0.000299 2.478666 -0.000027
H 2.065913 -1.311716 0.000054

C5H4N.H2O
E = -323.980827
C -1.635968 -1.179138 0.000000
C -0.618459 -2.147061 0.000000
C 0.671720 -1.662926 0.000000
C 0.992063 -0.319086 0.000000
N 0.000000 0.596541 -0.000000
C -1.269711 0.170327 0.000000
H 2.014304 0.059586 -0.000000
H -2.688054 -1.467055 0.000000
H -0.847860 -3.213634 0.000000
H -2.035874 0.948126 -0.000000
H 0.884260 2.336757 -0.000000
O 1.588260 3.015677 -0.000000
H 1.129276 3.862315 -0.000000

CN pathway, TS1
E = -416.893297
h 3.206475 0.146523 0.095635

o 2.388487 1.117440 -0.046323
c 1.621482 0.020270 -0.017579
c 0.209417 0.022214 -0.019031
n 2.531915 -1.005510 0.001827
c -0.555130 -1.197339 -0.048294
n -1.874585 -1.274752 -0.029382
c -2.584107 -0.105470 0.016748
c -1.946380 1.154570 0.036075
c -0.565559 1.239734 0.017314
h -0.050099 2.199340 0.031932
h -2.554464 2.063576 0.065828
h -3.672597 -0.192785 0.030975
h -0.018203 -2.150613 -0.100704
h 2.301341 -1.847606 0.528398

CN pathway, LM2
E = -416.929514
C 0.498937 -1.220196 0.000121
C -0.240797 0.022486 0.000013
C 0.572294 1.211798 0.000186
N 1.893034 1.244799 0.000047
C 2.567098 0.051860 -0.000188
C 1.882057 -1.183914 -0.000019
C -1.665637 0.085844 -0.000043
N -2.504673 1.103547 -0.000073
O -2.291288 -1.163837 -0.000060
H 0.078287 2.190150 0.000322
H 2.454905 -2.116368 0.000016
H -0.036835 -2.168102 0.000398
H -1.995188 1.985903 -0.000274
H 3.657841 0.102758 -0.000381
H -3.230951 -0.929339 0.000156

CN pathway, TS2
E = -416.918136
c -1.862855 1.187270 -0.020547
c -0.478353 1.208103 -0.042097
c 0.256132 -0.034339 -0.026705
c -0.571917 -1.216240 0.014359
n -1.894129 -1.235021 0.035837
c -2.557461 -0.041132 0.017176
c 1.686603 -0.107031 -0.027247
o 2.343675 1.150446 -0.040719
n 2.494889 -1.144242 -0.022964
h -0.090042 -2.199843 0.031674
h -2.425884 2.125307 -0.038868
h 0.061755 2.152578 -0.093480
h 1.938887 -2.001241 -0.055617
h -3.648600 -0.082613 0.033722
h 2.376269 1.477303 0.868582

CN pathway, LM3
E = -416.921744
C -1.881371 1.162918 0.065562
C -0.493739 1.213987 0.101189
C 0.263368 -0.025627 0.030063
C -0.544199 -1.210988 -0.046080
N -1.862057 -1.262092 -0.080938
C -2.550571 -0.067400 -0.033299
C 1.702836 -0.094467 0.011065
O 2.368346 1.127048 -0.130445
N 2.511162 -1.118841 0.107076
H -0.038405 -2.182255 -0.093893
H -2.462941 2.087501 0.131219
H -0.005213 2.180034 0.250152
H 1.961171 -1.961308 0.274422
H -3.639888 -0.126810 -0.064805
H 1.716831 1.782454 -0.407501

CN pathway, TS3
E = -416.896822
c 1.909248 1.145478 0.035714
c 0.520213 1.218357 0.034882
c -0.257804 0.002528 0.003338
c 0.526040 -1.204945 -0.034741
n 1.846398 -1.274256 -0.036295
c 2.550096 -0.100572 0.001922
c -1.687208 -0.094507 0.008863
o -2.381616 1.152290 -0.044458
n -2.419254 -1.181183 -0.056969
h -0.025543 -2.146682 -0.075631
h 2.507575 2.060447 0.069065
h 0.051261 2.204096 0.086603
h -2.730888 -1.632784 0.797897
h 3.639456 -0.180127 0.003508
h -1.742453 1.866773 -0.172786

CN pathway, LM4
E = -416.923772
C 2.558492 -0.076941 0.029417
C 1.896908 1.158735 -0.029203
C 0.507855 1.215594 -0.061094
C -0.246781 -0.021807 -0.023248
C 0.549452 -1.218688 0.012211
N 1.867813 -1.271033 0.040421
C -1.676025 -0.107818 -0.009369
N -2.376624 -1.213359 -0.064952
O -2.379880 1.111620 0.068864
H 0.016737 -2.172842 0.020016
H 2.482769 2.082263 -0.062894
H 0.025304 2.192171 -0.159378
H -3.367332 -0.971740 -0.002257
H 3.648153 -0.140045 0.055335
H -1.744318 1.813531 0.257701

CN pathway, TS4
E = -416.865924
o -2.898626 0.922068 0.889873
h -3.242550 -0.560755 -0.226931
c -1.446802 -0.419747 -0.372876
n -2.471651 -0.904492 -0.851224
c -0.093049 -0.194718 -0.238847
c 0.521538 1.075470 -0.528810
c 1.880849 1.216379 -0.314288
c 2.637803 0.141690 0.191713
n 2.074039 -1.059056 0.499681
c 0.777473 -1.211454 0.301261
h 0.347102 -2.181454 0.566005
h -2.788896 1.160020 1.820632
h -0.094438 1.899228 -0.885187
h 2.373028 2.168389 -0.527951
h 3.711181 0.237151 0.366343

CN pathway, LM5
E = -416.940759
o -5.090555 0.005210 0.114259
h -4.123886 0.007936 -0.081254
c -1.097207 0.013263 -0.172443
n -2.277299 0.008182 -0.251929
c 0.295666 0.029114 -0.079325
c 1.071961 1.260744 -0.025179
c 2.449206 1.156104 0.069397
c 3.079875 -0.102731 0.112118
n 2.359064 -1.282328 0.061407
c 1.049733 -1.196467 -0.028762
h 0.498404 -2.143152 -0.068130
h -5.116321 -0.042556 1.075891
h 0.573089 2.228995 -0.059756
h 3.061208 2.061987 0.111691
h 4.164195 -0.196035 0.186308

NCO pathway, TS
E = -416.314587
c -0.054131 -0.307719 -0.000018
h 2.028882 -1.622982 -0.000022
c 2.801086 0.024204 0.000005
n 2.964048 -1.200993 -0.000010
o 2.925558 1.194744 0.000022
c -0.567853 1.010442 -0.000020
c -1.944396 1.286591 -0.000006
c -2.838775 0.212832 0.000011
n -2.433261 -1.066310 0.000014
c -1.093901 -1.273520 0.000000
h -0.836645 -2.343766 0.000006
h 0.124139 1.864159 -0.000035
h -2.326808 2.314034 -0.000009
h -3.921733 0.384755 0.000022

O
E = -75.077162
O 0.000000 0.000000 0.000000

NH2
E = -55.872209
N 0.000000 -0.000000 0.143266
H -0.000000 0.807952 -0.501432
H -0.000000 -0.807952 -0.501432

CN
E = -92.720216
N 0.000000 0.000000 0.541845
C 0.000000 0.000000 -0.632152

C5H4N
E = -247.551143
C -1.179144 -0.812271 -0.000000
C 0.075785 -1.382457 -0.000000
C 1.252458 -0.654864 0.000000
N 1.200908 0.692694 0.000000
C 0.000000 1.285155 0.000000
C -1.215234 0.591728 -0.000000
H 2.240668 -1.116338 0.000000
H -2.163621 1.131522 -0.000000
H -2.095039 -1.405381 -0.000000
H 0.008448 2.377598 0.000000

[NAD-H], iso6
E = -416.264538
C 1.892518 1.142718 0.042124
C 0.503493 1.219816 0.044667
C -0.232653 0.026926 0.003839
C 0.468033 -1.190441 -0.040755
N 1.803546 -1.272262 -0.041318
C 2.491825 -0.122964 0.000785
C -1.715982 0.071128 0.019338
O -2.363248 1.121149 -0.116177
N -2.402987 -1.122371 0.040429
H -0.080755 -2.131944 -0.089083
H 2.508782 2.040875 0.074123
H -0.019063 2.175355 0.077212
H -3.330797 -0.958293 0.450852
H 3.580497 -0.215850 0.002559

NCO
E = -168.019808
N 0.009369 1.271717 0.000000
C 0.000000 0.040182 0.000000
O -0.008198 -1.142889 -0.000000

[NAD-CN]-
E = -324.007113
N 1.120721 -1.167813 -0.018131
C 1.830532 0.026982 0.003315
C 1.156701 1.216695 0.002158
C -0.268878 1.262144 -0.003306
C -0.959628 0.029652 0.000339
C -0.285936 -1.174532 0.002919
O -2.331203 0.089439 0.002311
H -0.774809 -2.145274 -0.002252
H 1.733448 2.140754 0.005583
H -0.821848 2.197290 -0.001547
H 2.914128 -0.054145 0.007712
H -2.697386 -0.803480 0.001566
H 1.614302 -2.041612 0.064812
